# Neurocognitive and Somatic Components of Temperature Increases during g-Tummo Meditation: Legend and Reality

**DOI:** 10.1371/journal.pone.0058244

**Published:** 2013-03-29

**Authors:** Maria Kozhevnikov, James Elliott, Jennifer Shephard, Klaus Gramann

**Affiliations:** 1 Psychology Department, National University of Singapore, Singapore; 2 Martinos Center for Biomedical Imaging, Department of Radiology, Harvard Medical School, Charlestown, Massachusetts, United States of America; 3 Department of Psychological and Brain Sciences, University of California Santa Barbara, Santa Barbara, California, United States of America; 4 Division of Social Science, Harvard University, Cambridge, Massachusetts, United States of America; 5 Biological Psychology and Neuroergonomics, Berlin Institute of Technology, D-Berlin, Germany; 6 Swartz Center for Computational Neuroscience, University of California San Diego, La Jolla, California, United States of America; St. Joseph’s Hospital and Medical Center, United States of America

## Abstract

Stories of g-tummo meditators mysteriously able to dry wet sheets wrapped around their naked bodies during a frigid Himalayan ceremony have intrigued scholars and laypersons alike for a century. Study 1 was conducted in remote monasteries of eastern Tibet with expert meditators performing g-tummo practices while their axillary temperature and electroencephalographic (EEG) activity were measured. Study 2 was conducted with Western participants (a non-meditator control group) instructed to use the somatic component of the g-tummo practice (vase breathing) without utilization of meditative visualization. Reliable increases in axillary temperature from normal to slight or moderate fever zone (up to 38.3°C) were observed among meditators only during the Forceful Breath type of g-tummo meditation accompanied by increases in alpha, beta, and gamma power. The magnitude of the temperature increases significantly correlated with the increases in alpha power during Forceful Breath meditation. The findings indicate that there are two factors affecting temperature increase. The first is the somatic component which causes thermogenesis, while the second is the neurocognitive component (meditative visualization) that aids in sustaining temperature increases for longer periods. Without meditative visualization, both meditators and non-meditators were capable of using the Forceful Breath vase breathing only for a limited time, resulting in limited temperature increases in the range of normal body temperature. Overall, the results suggest that specific aspects of the g-tummo technique might help non-meditators learn how to regulate their body temperature, which has implications for improving health and regulating cognitive performance.

## Introduction

The g-tummo meditative practice targeted at controlling “inner energy” is described by Tibetan practitioners as one of the most sacred spiritual practices in the Indo-Tibetan traditions of Vajrayana Buddhism and Bon. It is also called “psychic heat” practice since it is associated with descriptions of intense sensations of bodily heat in the spine [1]–[3]. Little is known about the specifics of the g-tummo technique. Monasteries maintaining an extensive practice of g-tummo are quite rare and located mostly in the remote Chinese provinces of Qinghai and Sichuan (also known as eastern Tibet). Eyewitness accounts describe g-tummo practitioners as being able to generate sufficient heat to dry wet sheets wrapped around their naked bodies, producing a visible amount of steam, while sitting or walking in the freezing cold of the Himalayas [4], [5].

The only attempts to study the physiological effects of g-tummo have been made by Benson and colleagues [6], [7] who researched Indo-Tibetan Yogis in the Himalayas and in India. The authors reported that three g-tummo meditators showed a dramatic increase of up to 8.3°C in peripheral body temperature (fingers and toes), more modest skin temperature increases of 1.9°C in the navel and lumbar regions, and no increase in rectal temperature. Unfortunately, these findings have subsequently been distorted in reports in other sources, possibly due to confusion between Fahrenheit and Centigrade scales or lack of clear specification regarding the anatomical sites of temperature measurement, leading to general claims of temperature increases during g-tummo ranging from “… up to 15 degrees only within a few moments of concentration” [3] to “17 degrees in peripheral body temperature” [8].

There is currently no evidence, however, indicating that temperatures are elevated beyond the normal range during g-tummo meditation. The visual effect of steaming sheets reported by eye-witnesses of the g-tummo ceremony cannot be taken as evidence of elevated body temperature. Wet sheets wrapped around a practitioner’s body would steam and dry due to the significant temperature difference between the wet sheets (heated by a human body) and the cold air outside, even if the practitioners simply maintain their normal body temperature. As impressive as the peripheral body temperature increases during g-tummo meditation reported by Benson et al. might seem, they were in the range of normal body temperature (finger and toe temperatures increased from 22°C to 33°C). Furthermore, they did not exceed the peripheral body temperature increases reported in clinical studies of (non-meditating) individuals who were able to increase hand or finger temperature by up to 11.7°C during biofeedback alone or in combination with hypnosis, mental imagery, or autogenic training [9]–[11]. Subsequent clinical research, however, reported that such peripheral temperature increases were primarily mediated by somatic (e.g., altered respiration and/or tensing and contracting of muscles) but not cognitive factors [12].

The g-tummo practice involves both somatic and neurocognitive components. The somatic component involves specialized breathing techniques as well as isometric exercises (i.e. exercises performed in static positions, rather than incorporating a range of motion) involving muscle tensing and contraction. The neurocognitive component involves meditative visualization requiring the generation and maintenance of mental images of flames at specific locations in the body accompanied by intense sensations of bodily heat in the spine. The questions remain as to whether the g-tummo practice is indeed associated with elevated body temperature, and whether these temperature increases are due to cognitive (e.g., attention, mental imagery) or merely somatic, components of the practice. Thus, the goals of the current research were 1) to explore systematically the temperature increases and neural (EEG) activity associated with g-tummo practices; and 2) to investigate the contribution of neurocognitive versus somatic components of the g-tummo practice to the temperature increases associated with the practice. First, we conducted a study in remote monasteries of eastern Tibet with ten expert meditators performing g-tummo practices while their axillary body temperature and electroencephalographic (EEG) activity were measured. Second, to further investigate the contribution of somatic versus neurocognitive components of the g-tummo practice, we conducted an additional study with eleven Western participants (non-meditators) instructed to use the somatic component of the g-tummo practice without utilization of meditative visualization.

If it is true that g-tummo meditators can elevate their body temperature beyond normal as a result of g-tummo meditation, this would have a number of important theoretical and practical implications. Recent studies report that raising body temperature might be an effective way to boost immunity and treat infectious diseases and immunodeficiencies [13]–[15] as well as to induce synaptic plasticity in the hippocampus [16]. It has been long recognized that increased body temperature (in the zone of a slight fever) is associated with higher alertness, faster reaction time, and better cognitive performance on tasks such as visual attention and working memory [17]–[19]. Thus, an understanding of the mechanisms underlying body temperature increases during g-tummo practice could lead to the development of effective self-regulatory techniques in “ordinary” individuals (e.g., non-meditators) to regulate their neurocognitive functions and fight infectious diseases.

## Methods

### Study 1

In order to access experienced g-tummo meditators, the first author travelled to Gebchak nunnery, which is the only nunnery in Tibet with a tradition of extensive g-tummo practice. Gebchak nunnery is located close to Nangchen town, in the Qinghai province of China (above 4200 m altitude) and is very remote and isolated. Gebchak Wangdrak Rinpoche, the abbot of Gebchak nunnery, helped with all the logistical arrangements and the recruitment of meditators (N = 10, 7 females) from Gebchak nunnery and other local monasteries, such as Chobdrak monastery (Barom Kagyu linage), Yachengar monastery (Nygma linage), and Kala Ring-go (Karma Kagyu). One meditator was tested in her hermitage, one in the guest room of Gebchak nunnery, and the rest were tested in a typical unheated mud-and-brick house in Nangchen. The room temperature in these locations during January, when the testing was done, is usually around 0°C. A Tibetan-English interpreter translated all instructions into Tibetan before each testing sessions began. The research was approved by National University of Singapore institutional review board. All the participants provided their written consent to participate in the study.

Participants ranged in age from 25 to 52 years, and their g-tummo meditation experience ranged from 6 to 32 years. The monasteries the participants were recruited from vary slightly in the emphasis they place on different stages of the g-tummo practice. Some, but not all, of these monasteries test their practitioners’ capabilities at the end of a three-year retreat with a ceremony where the practitioners dry wet sheets. As a testament to the importance of the g-tummo practice at Gebchak nunnery, this ceremony is held annually, at dawn, and all of the experienced practitioners walk slowly for a few hours around the nunnery complex in −25°C to −30°C weather, wearing only short skirts and shoes and a wet sheet draped around their naked torsos.

#### Two types of g-tummo practice

The g-tummo practice is characterized by a special breathing technique, “*the vase*”, accompanied by isometric muscle contractions, where after inhalation, during a period of holding their breath (apnea), the practitioners contract both abdominal and pelvic muscles so that the protruding lower belly takes the shape of a vase or pot [1].

Oral tradition, as confirmed by our extensive interviews with g-tummo practitioners, differentiates between at least two main types of g-tummo practice, *Forceful Breath (FB)* and *Gentle Breath (GB)*. These types of g-tummo practice differ not only in terms of the breathing technique involved, but also in their goals and the content of visualization. Although both FB and GB are based on the “vase” breathing technique, FB is forceful and vigorous, while GB is gentle and without any strain. Whereas the goal of FB is to raise “psychic heat”, the goal of GB is to maintain it. During FB, attention is focused on visualizing a rising flame that starts below the navel and with each breath rises up to the crown of the head, whereas GB is accompanied by visualization of the entire body being filled with a surging sensation of bliss and heat. It is physically difficult for practitioners to use the complex FB technique for extended periods of time or while walking around; therefore it is GB that is used during the ceremony of drying wet sheets.

#### Procedure

We recorded EEG activity of the meditators as well as their peripheral (left fifth finger) and core body temperature (left armpit) during g-tummo practices in four conditions:1) *Baseline FB (BFB),* during which the participants were asked to breathe and perform (specifically, contract abdominal and pelvic muscles as well as maintain hand and body positions) exactly the same way they perform during *FB* but without meditative visualization; 2) *Baseline GB (BGB)*, during which the participants were asked to breathe and perform exactly the same way they perform during *GB* but without meditative visualization; 3) *Meditation FB (MFB)*, during which the participants were asked to perform their usual *FB* meditation practice, including vase breathing and visualization; and 4) *Meditation GB (MGB)*, during which the participants were asked to perform their usual *GB* meditation practice including vase breathing and visualization. Participants’ eyes remained opened during all the conditions.

Due to differences in the meditators’ experience in FB and GB practices, and their time availability, only four participants completed all conditions in the following continuous sequence: BFB, BGB, MFB, and MGB. Two of the remaining participants performed the BFB followed by MFB conditions, and four completed BGB followed by MGB. The duration of FB and GB varied between participants; while a few were not able to perform FB continuously for more than 6–7 min, others were able to continue for up to 50 min. Thus, we asked the participants to perform each specific practice for as long as they felt comfortable; however, both baseline conditions (BFB and BGB) were limited to 10 min.

Most experiments were conducted during the first half of the day (8∶00 am−3∶00 pm). During data acquisition, all participants wore a wireless sensor headset (B-Alert Headset Model 600B, ABM), which consists of head and host units for bi-directional transmission of digitized physiological signals, and a sensor headset cap with sensors at Fz, Cz, POz, F3, F4, C3, C4, P3, and P4 and referenced to linked mastoids. The signals were communicated using a 2.4 to 2.5 GHz radio transmitter. A standard Class 1 Bluetooth dongle was used as the receiving base unit affixed to the PC workstation. EEG was recorded with a digitization rate of 256 Hz.

We also measured apnea duration (in sec) during BFB and MFB by recording the breathing sounds with a microphone placed near the practitioners during their practice. Both inhalation and exhalation of the “forceful” type of vase breathing are characterized by specific sounds. Inhalation is relatively long and loud. Exhalation is fast and forceful and is accompanied by distinct “huh!” sound. During data analysis, the waveform of the audio signal was analyzed using audio-analysis software (Goldwave v5, Goldware, Inc) to determine the beginning of each inhalation and exhalation. The time period between each inhalation and exhalation was then measured, and the average was computed. We did not measure the apnea duration during GB since breathing is more natural during this practice.

Participants’ body temperature was measured using small disk thermometers of 5 mm in diameter, attached to the body with adhesive tape. The thermometer was connected to a computer through a USB high precision 8-channel temperature measurement device from the Measurement Computing Corporation. The temperature data were sampled at a rate of 100 Hz. The sensor’s operating temperature range is from −35°C to 120°C; the maximum error is 0.001°C. One thermometer was placed on the left fifth finger to measure peripheral body temperature and another one under the armpit to measure core body temperature at least 10 min prior to taking any measurements to allow the temperature to stabilize. Although not as precise as an internally taken rectal or oral measurement of core body temperature [20], axillary measurements are less intrusive. Importantly, they are not affected by muscle contractions (e.g., anal sphincter), or the airflow through the mouth, during the vase breathing.

### Study 2

#### Participants and procedure

Eleven Western participants (10 females) who had experience in breathing and isometric exercise in different branches of yoga (e.g., hatha yoga, kundalini yoga) and martial arts (e.g. kung fu) participated in this study (Mexp = 13. 6 years, range from 7−30 years). The participants did not have any experience in Tibetan meditation practices. Their age ranged from 46 to 70 years old, Mage = 52.64. The experiments were conducted in one of the studios in the Shakti Yoga School (Mapplewood, NJ) during the first half of the day (9∶00 am – 3∶00 pm).

First the participants, in groups of 3–4, were given detailed instructions on how to perform BFB (vase breathing with corresponding isometric exercises) followed by a 30 min training session. Then, all the participants were tested individually in a 45–60 min session, during which they were requested 1) to rest for 15–20 min; 2) to perform BFB as long as they felt comfortable; 3) to rest again for another 15–20 min. During the session, participants’ axillary temperature was measured using a small disk thermometer (Measurement Computing Corporation), attached to the left armpit of the participants with adhesive tape, similar to the one used in Study 1. In addition, similar to Study 1, we also measured participants’ apnea duration during BFB exercises by recording the breathing sounds with a microphone placed nearby.

Two additional Western participants (2 females, Mage = 46.3) were invited on a different occasion. One of the participants had extensive experience in hatha yoga; she was tested in a session similar to the other participants, however, she was asked to perform BFB twice. That is, after completion of her first BFB session, she was asked to rest until she felt comfortable enough to continue with the second round of BFB, followed by a final 15 min rest. The second participant had 7 years experience in g-tummo practices, and she was tested in a session similar to the first participant, however, instead of a second round of BFB, she was asked to perform MFB.

## Results

### Study 1

#### Temperature changes

**Figure 1:**
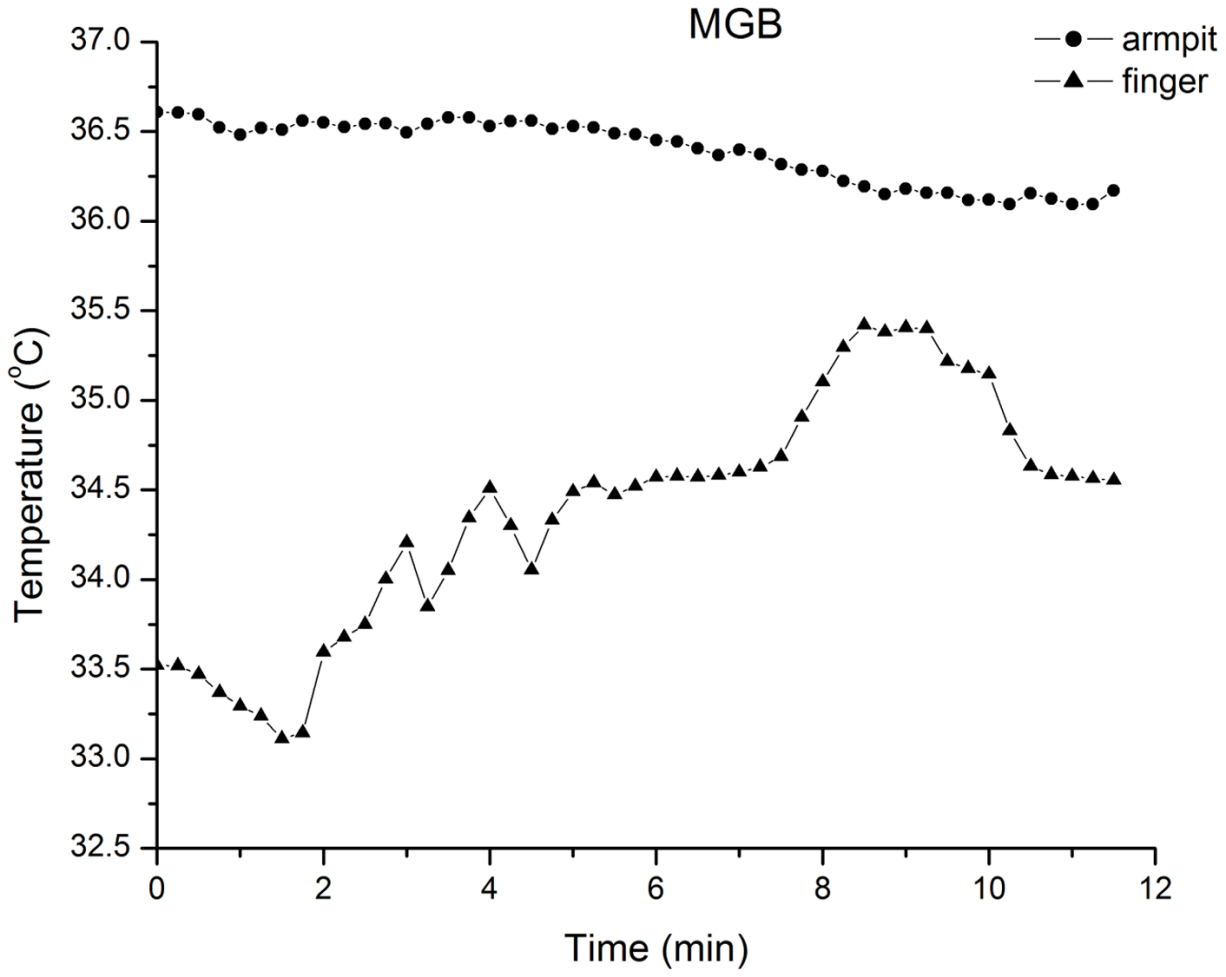
Representative temperature data taken at armpit and left fourth finger of one of the Study 1 participants during BGB and MGB.

**Figure 2:**
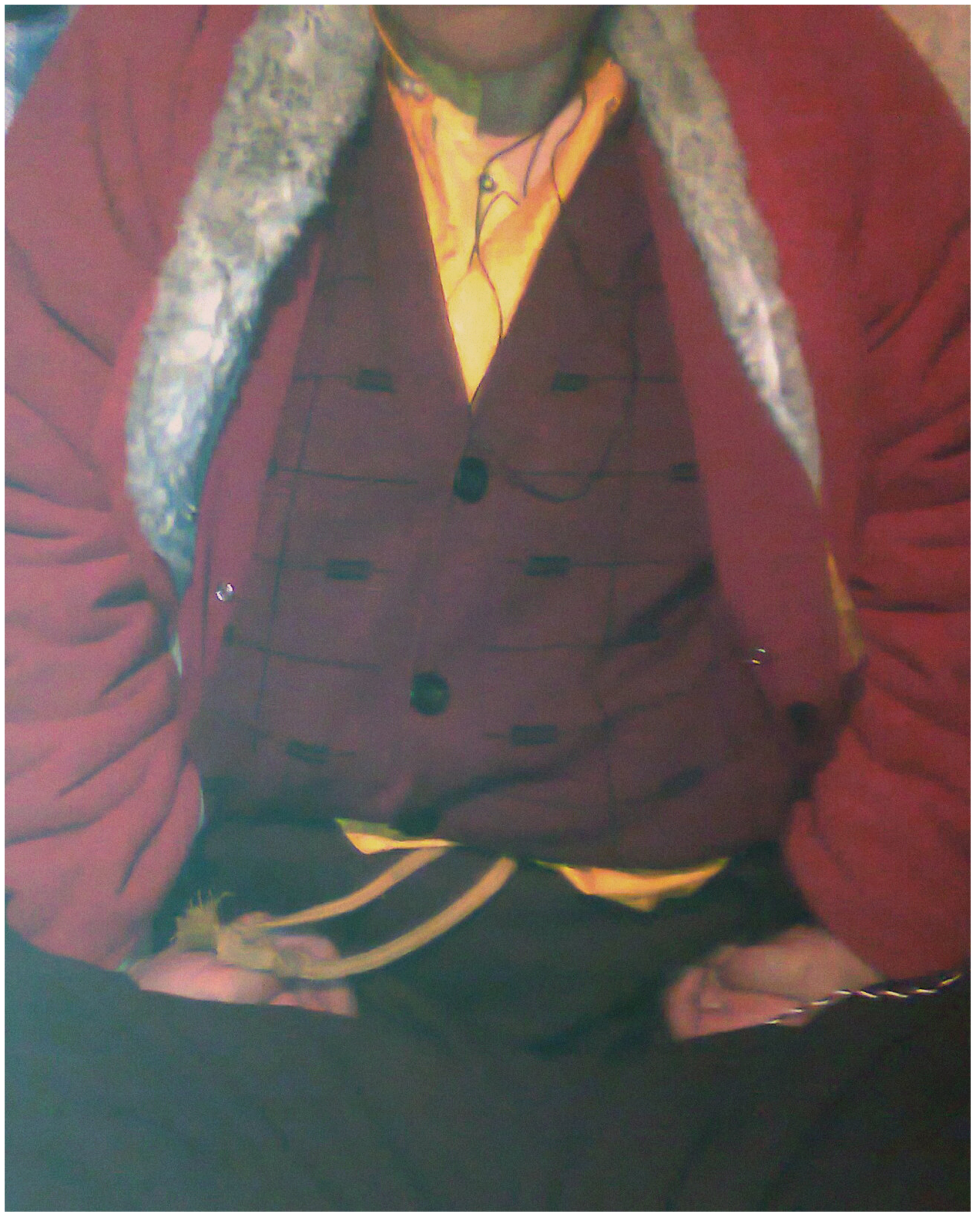
Hand position during g-tummo meditation.

For each participant, temperature data were averaged over 15 sec intervals. The results indicated peripheral (finger) temperature increases between 1.2°C to 6.8°C during different conditions (see **Figure 1** for a representative participant measured at the left fifth finger during MGB; while the peripheral temperature underwent pronounced changes, axillary body temperature remained constant at around 36.6°C). The peaks of the peripheral temperature increases were associated with changes in hand mudra positions (symbolic gestures used in meditation), e.g., tensing the hand muscles as well as pressing the fists against the inguinal crease (on the femoral artery) during particular meditative periods (**Figure 2**). This suggests that the peripheral temperature increases are primarily the result of increased peripheral blood flow due to peripheral muscular action (and proximity of the femoral artery) rather than a result of psychological (caused by meditation) or physiological (caused by breathing or isometric techniques) states. Since hand movements constitute an integral part of g-tummo, it was impossible to eliminate the effect of these factors on peripheral temperature increases. Thus, in all further analyses we focus on core body temperature (CBT) data from the armpit sensors only.

**Table 1** presents the data for axillary temperature for each of the participants at the beginning and at the end of each condition (BFB, BGB, MFB, MGB) they performed. Parametric statistics were used to analyze the temperature data despite the small sample size used in the current study, since the temperature data in the population fit a normal distribution [21]. The average initial CBT of all the participants was 36.49°C (SD = 0.21), which is not significantly different from the normal axillary temperature of 36.6°C in the healthy population [one sample two-tailed *t*(9) = 1.71, *p* = 0.12]. The average temperature at the end of BFB was 36.9°C (SD = 0.32), which is only marginally above the normal axillary temperature: *t*(5) = 2.52, *p* = .053. During BFB, the maximum CBT increase was 1.14°C (participant #3) and the maximum temperature reached was 37.45°C (participant #4). By the end of MFB, the average temperature increased to 37.6°C (SD = 0.52), which is significantly above the normal axillary temperature [*t*(5) = 4.77, *p* = .005]. The maximum CBT increase from the beginning of the experiment to the end of MFB was 2.2°C (participant #3), and the maximum temperature reached was 38.30°C (participant #5). No elevation in CBT was observed among the four participants who performed only the GB practice. The final average CBT of those four participants after BGB and MGB was 36.42°C (SD = 0.14).

**Table 1:** Initial (t0) and final (t1) CBT for each of the four practices.

Participant	BFB		BGB		MFB		MGB	
	t0	t1	t0	t1	t0	t1	t0	t1
1	36.56	36.79	36.79	37.00	37.03	37.44	37.44	37.46
2	36.49	36.66	36.68	36.77	37.09	37.12	37.05	37.09
3	36.00	37.14	37.16	37.29	37.30	38.21	38.21	38.03
4	36.56	37.45	37.45	37.49	37.48	37.50	37.49	37.49
5	36.82	37.00	–	–	37.07	38.30	–	–
6	36.53	36.58	–	–	36.86	37.12	–	–
7	–	–	36.54	36.46	–	–	35.98	36.25
8	–	–	36.49	36.49	–	–	36.51	36.5
9	–	–	36.56	36.56	–	–	36.57	36.57
10	–	–	36.31	36.36	–	–	36.35	36.36

**Figure 3:**
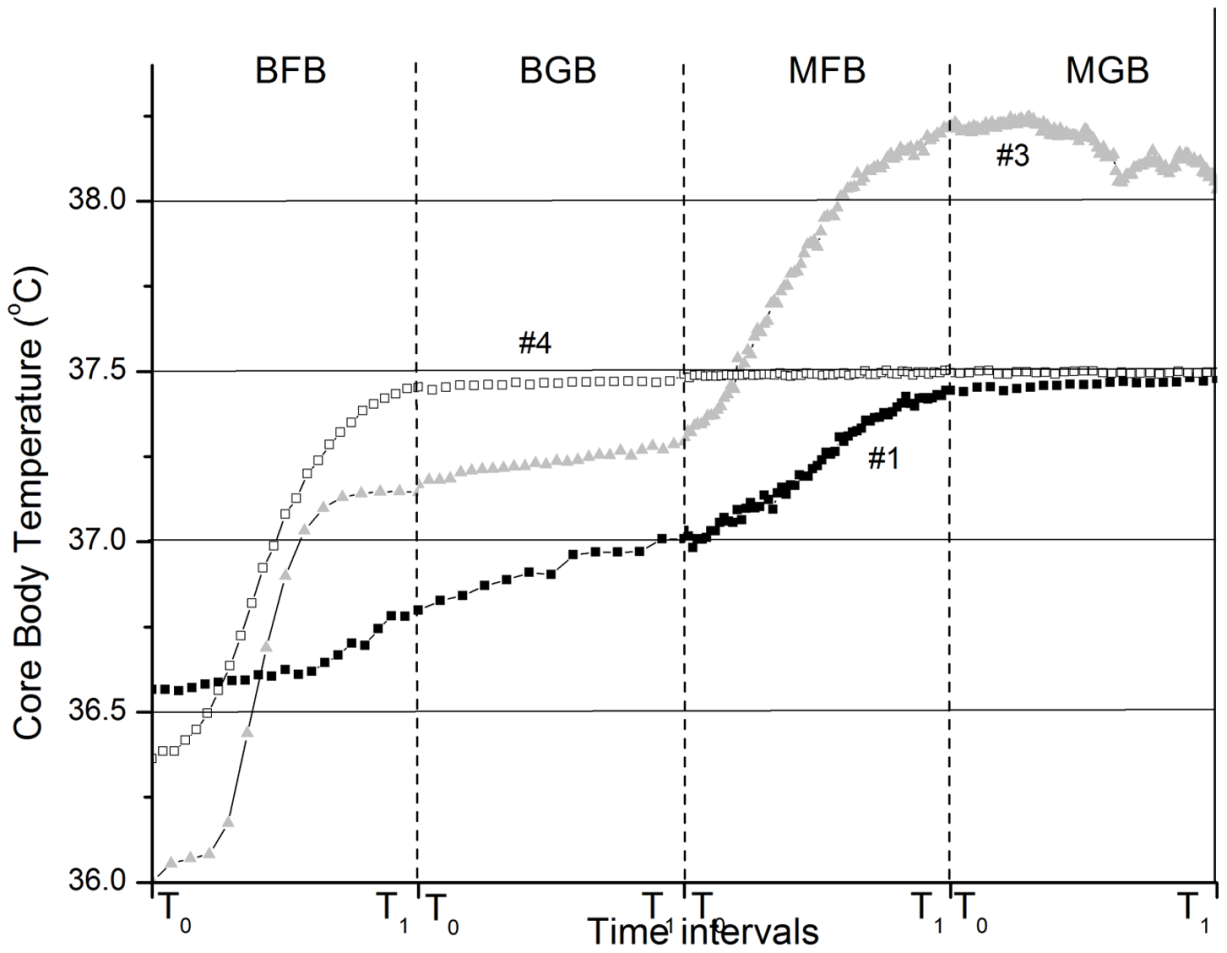
CBT increases for Study 1 participants #1, #3, and #4 during BFB, BGB, MFB, and MGB performed in a continuous sequence. Since the duration of each of the four practices varied from participant to participant, to simplify the figure presentation, the duration of each practice is rescaled from 0 to 1, with t0 the starting point of each practice, and t1 the ending point.

**Figure 3** shows the CBT increases for three representative participants who completed the sequence of all four conditions (BFB, BGB, MFB, and MGB).

During FB (either BFB or MFB), participants’ CBTs typically exhibited a step-like pattern, with a period of steady temperature increase followed by a plateau or equilibrium phase corresponding to the “temperature saturation point”, above which the participants were not able to raise their CBT further despite their efforts to continue with FB. This pattern of CBT increases is very similar to that usually observed during induction of systemic hyperthermia (i.e., deliberate heating of a patient’s body to achieve an elevated core temperature for therapeutic purposes), where the equilibrium phase indicates the beginning of heat losses due to physiological mechanisms (e.g. vasodilation, evaporation) limiting the rate of heating that can be achieved, and thus protecting the body from excessively high temperatures [22]. For example, one of the six participants who performed FB reached the equilibrium phase very quickly during the end of BFB (participant #4, Figure 3) and despite her continuous efforts, she was not able to raise it further during MFB, whereas two participants reached the equilibrium only at the end of the MFB practice (e.g., participant #1). Three other participants exhibited two equilibrium plateaus, one at the end of BFB and another at the end of MFB (e.g., participant #3, Figure 3), suggesting that the meditative visualization they performed might have dampened the physiological mechanisms leading to the heat losses.

**Figure 4:**
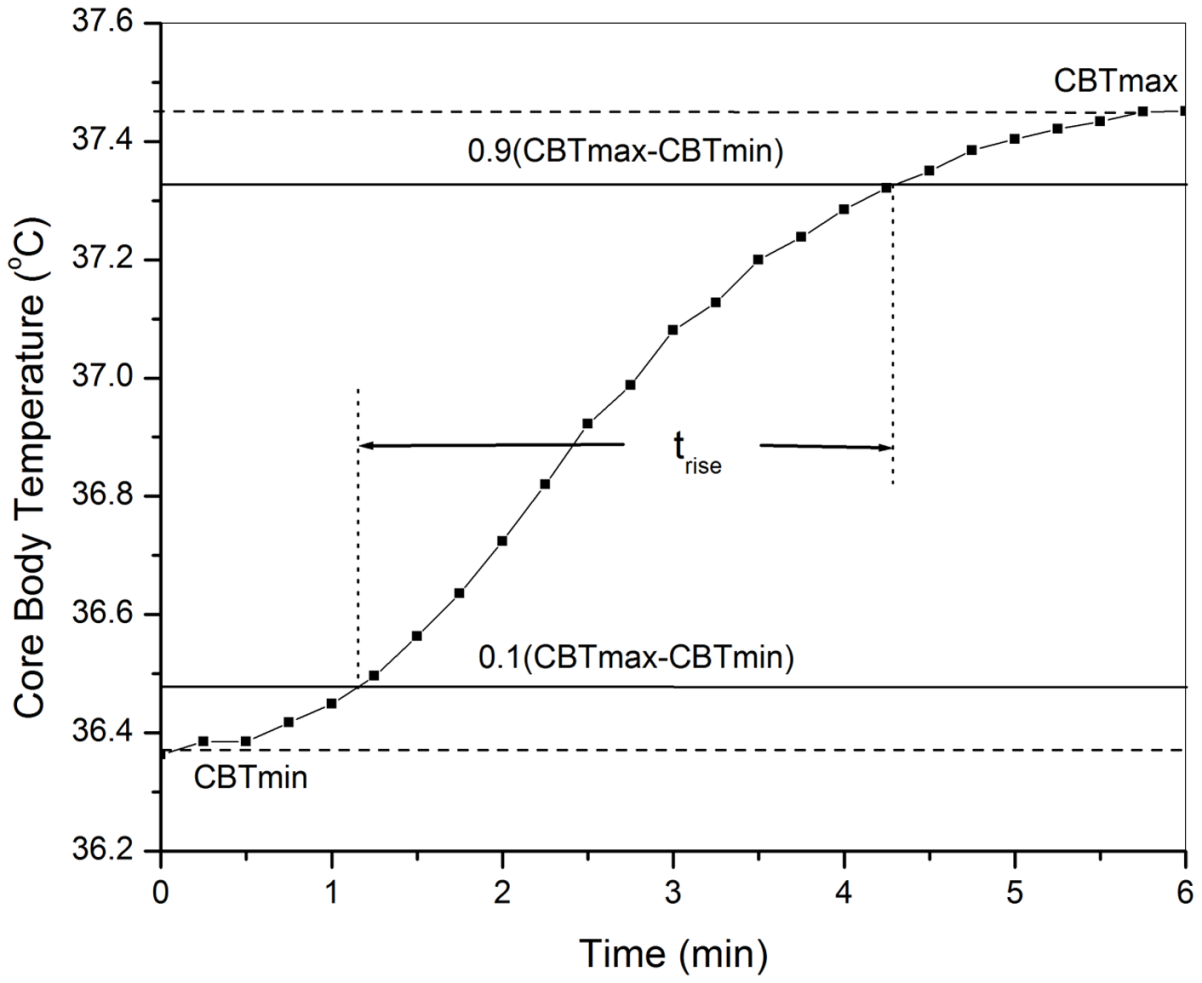
CBT rise time for Study 1 participant #4 during BFB. The rise time is taken as the time during which the CBT rises from 10% to 90% of its maximum value.

To quantify the effective period of steady CBT increases for those participants who exhibited temperature equilibrium phases during either BFB or MFB, we defined the *rise time* (ΔTr) as the time taken for the CBT to rise from 10% to 90% of its final value (see **Figure 4**). This is similar to the definition of rise time in signal control theory, describing the transition time for a signal that changes from one level to another [23], [24]. For all the BFB and MFB conditions where the participant CBT did not reach equilibrium phase by the end of the condition, the rise time was taken as time for the CBT to increase from 10% to 100%.

Furthermore, to quantify the rate of steady CBT increases, we conducted linear regression analyses (CBT increases were regressed on the rise time for BFB and MFB and on the overall condition time for BGB and MGB) for each participant separately. All linear regressions for each participant for each of the above four time periods were significant (all R squares >0.88, all *p*s <0.001). In order to account for possible variations between participants, we treated regression coefficients as random variables, and used a linear mixed effect (MIXED) model to estimate the population regression lines. The regression intercepts and slopes δ (representing *the rate* of axillary temperature change per unit time) are presented in **Table 2** for BFB, BGB, MFB, and MGB separately. The regression slopes were significantly different from zero for BFB and MFB (*p*s <0.05) only. The variance of intercept, the variance of slopes, and the covariance between the intercepts and the slopes were non-significant.

**Table 2:** Regression coefficients δ, intercepts, duration ΔT and rise time ΔTr.

	δ °C/min (SD)	Intercept °C(SD)	ΔT min (SD)		ΔTr min (SD)
BFB	0.146(0.072)	36.09(0.26)	5.91 (1.37)	5.71 (1.31)	
BGB	0.011(0.008)	36.82(0.21)	6.37 (1.16)	–	
MFB	0.030(0.009)	37.02(0.32)	19.41 (15.69)	18.76 (15.14)	
MGB	0.002(0.005)	37.02(0.43)	21.50 (13.10)	–	

In summary, the results suggest that although CBT increases during BFB were not as dramatic as during MFB, participants were able to produce body heat, utilizing only the somatic component of the FB practice (breathing and isometric techniques). However, the meditators were able to reach an elevated CBT, significantly above the normal axillary temperature, only during MFB practice. As for GB practice, consistent with practitioners’ reports that it is used to maintain (but not to increase) body heat, no significant changes in participants’ CBT were observed during either GB baseline or meditation.

#### EEG analysis

After a high-pass filter at 1 Hz was applied to the EEG data, the files were epoched into 1-second intervals. The five-minute periods for each of the four conditions for each participant that had the least amount of noise were isolated and any epoch that had values of +/−200 µV or more were excluded from the data. In order to rule out differences due to the time period selected, we did an additional analysis that compared a one-minute interval at the start of MFB with a one-minute interval at the peak temperature for each participant. There were no significant differences between these two time periods in the power of the frequency ranges examined. Thus, in our final analysis we included 5 min intervals that had the least amount of noise. Subsequently, eye-blinks, muscle activity, and movement artifacts were rejected by visual inspection. Of the original 300 epochs, there remained, on average, 162+/−73 epochs. EEG frequencies were calculated for each condition and for each electrode using Welch’s method as implemented in the EEGlab toolbox for Matlab, with a window of 256. This function returns 10×log10(µV^2^), which results in units of dB. Standard frequency ranges were then defined: theta (4.5–7.5 Hz), alpha (8.5–12.5 Hz), beta (13–25 Hz), and gamma (35–45 Hz), and power values within those ranges were averaged. Oscillatory EEG data has been shown to be normally distributed [25], and therefore standard parametric tests were used.

The most pronounced differences in brain activity for meditative as compared to baseline condition were observed for Forceful Breath. There was a significant increase in the alpha power band during FB meditation (BFB_α_ = 3.88 dB, MFB_α_ = 5.28 dB; *F*(1,5) = 15.9, *p* = .01) revealing a dominantly parieto-occipital topography. A significant power increase was also observed for beta (BFB_β_ = 0.44 dB, MFB_β_ = 1.01 dB; *F*(1,5) = 20.59, *p*<.01) as well as a strong tendency toward a significant power increase in the gamma frequency band (MBF_γ_ = −2.06 dB, BFB_γ_ = −2.83 dB; *F*(1,5) = 5.3, *p* = .06). Both beta and gamma increases were most pronounced at lateral frontal sites. There was no significant difference in theta frequency band between BFB and MFB (BFB_θ_ = −8.04 dB, MFB_θ_ = −7.73 dB).

For Gentle Breath, only beta activity demonstrated a significant increase in power between BGB and MGB (BGB_β_ = −0.14 dB, MGB_β_ = 0.96 dB; *F*(1,7) = 7.87, *p*<.05). In contrast to FB, there was no significant difference between BGB and MGB in alpha (BGB_α_ = 4.17 dB, MGB_α_ = 5.51 dB; *F*(1,7) = 2.45, *p = *0.16) or in gamma power (MBF_γ_ = −3.41 dB, BFB_γ_ = −2.22 dB; *F*(1,7) = 2.23, *p = *0.18). Also, there was no difference in theta power (BGB_θ_ = −4.05 dB, MGB_θ_ = −4.75 dB).

**Figure 5:**
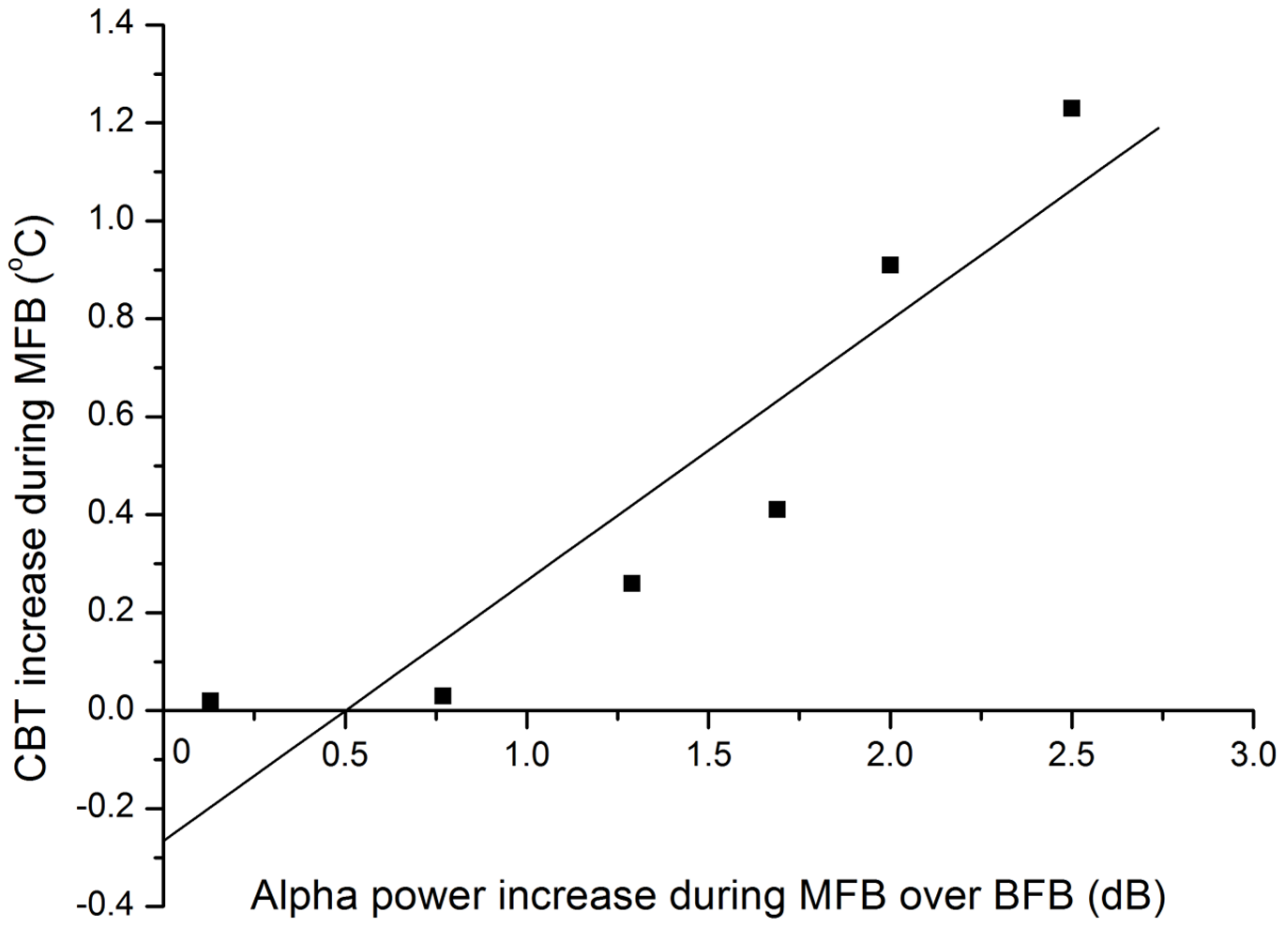
CBT increases during FB meditation in Study 1as a function of alpha power increases during MFB over BFB (solid line represents regression).

To investigate potential relationships of power in distinct frequency bands and CBT increases, we correlated the increases in alpha, beta, and gamma power during MFB over BFB with the magnitude of the CBT increase during MFB. Interestingly, there was a significant linear relationship (**Figure 5**) between the increase in alpha power during MFB over BFB and the CBT increases during MFB (R square = 0.82, *p*<0.01). None of the other relationships were significant.

To investigate whether there was a similar relationship between increases in alpha power and CBT increases during BFB, we conducted an additional analysis that compared alpha power of the practitioners at a one-minute interval at the start of BFB with a one-minute interval at the end of BFB. There were no significant differences between these two time periods in the alpha power: *F*(1,5) = 1.95, *p = *0.22, suggesting that, in contrast to MFB, CBT increases during BFB are not related to the alpha band activity.

In summary, the results suggest that besides differences in CBT changes, Forceful and Gentle Breath meditation are associated with divergent brain state dynamics. Furthermore, the higher the increases in alpha power developed by participants during FB meditation, the larger their increases in CBT during FB meditation, while the CBT increases during BFB were achieved without any changes in alpha power. This suggests that different mechanisms may be affecting CBT increases during MFB versus BFB, and that meditative visualization characterized by significant increases in alpha power might uniquely contribute to overall CBT increases beyond the contribution of the vase breathing technique. Further analyses were conducted to investigate different factors contributing to CBT increases during BFB and MFB.

#### Factors contributing to CBT increases

The CBT increases (Δ*t*) during BFB and MFB could be described according to a linear regression equation, as Δ*ti* ≈ δ*i*ΔTr*i*, where δ*i* represents the regression coefficient (the rate of CBT change per unit time) and ΔTr*i*, represents the rise time of the CBT for condition *i* (mean δs and ΔTrs for each condition *i* are given in **Table 2**). Further analyses were conducted to investigate factors contributing to the overall CBT increase (Δ*t*) by affecting 1) the rate of CBT increase (δ), and 2) the CBT rise time (ΔTr).

First, we computed the average length of time each participant held his/her breath (apnea duration) during BFB [Mean = 1.58(0.52) min, range 1.30–2.50 min] and MFB [Mean = 1.50(0.45) min, range 1.15–2.30 min], and correlated the apnea duration during BFB and MFB with δBFB, δMFB, ΔTBFB, and ΔTMFB. There was a significant positive correlation between the apnea duration during BFB and δBFB (r = 0.91; *p*<.05). There was also a trend toward a correlation between the apnea duration during MFB and δMFB (r = 0.72; *p* = 0.1). All other correlations were non-significant. This suggests that apnea duration, during which the practitioners hold their breath while concurrently maintaining isometric muscle tension, is related to the rate of the CBT increase, thus contributing to the overall CBT increases during g-tummo practices.

Second, we correlated the increases in alpha power during MFB over BFB with δBFB, δMFB, ΔTrMFB, and ΔTrMFB. There was a significant linear relationship between the increase in alpha power during MFB over BFB and the ΔTrMFB (r = 0.94, *p*<0.01); that is, those who showed a greater increase in alpha power during meditation were capable of prolonging their CBT rise time without reaching equilibrium, thus reaching higher overall CBT increases. None of other relationships were significant.

In addition, for those participants who performed FB, we correlated their age (Mage = 44. 16 (SD = 5.49) y.o., range 38 −51 y.o.) as well as their experience in g-tummo (Mexp = 12. 50(SD = 3.93) years, range 19–30 years) with their δBFB, δMFB, ΔTrMFB, and ΔTrMFB. None of the relationships were significant. However, the lack of correlations is not surprising, taking into account that all the practitioners who performed FB were of similar middle age and all of them had extensive g-tummo experience, including at least three three-year g-tummo retreats.

In summary, our findings indicate that the two parameters, apnea duration and increases in alpha power achieved during meditative visualization are significant predictors of the overall CBT increases during FB practice. The apnea duration is significantly related to the rate of CBT increase**.** The increase in alpha power developed during FB meditation is related to the CBT rise time, that is, it predicts how long the meditators are capable of sustaining their CBT increases without reaching equilibrium.

### Study 2

The CBT changes for all the participants showed a similar pattern, which involved 1) a 10 min period of thermometer heating under the arm until it reflected an accurate axillary temperature; 2) a baseline (rest) period of relatively constant CBT until the beginning of BFB; 3) a steady CBT increase during BFB, followed by a plateau (equilibrium) where the temperature was maintained as long as the participant continued with BFB; and 3) a cool-down phase to the baseline temperature during the final 20 minutes.

**Figure 6:**
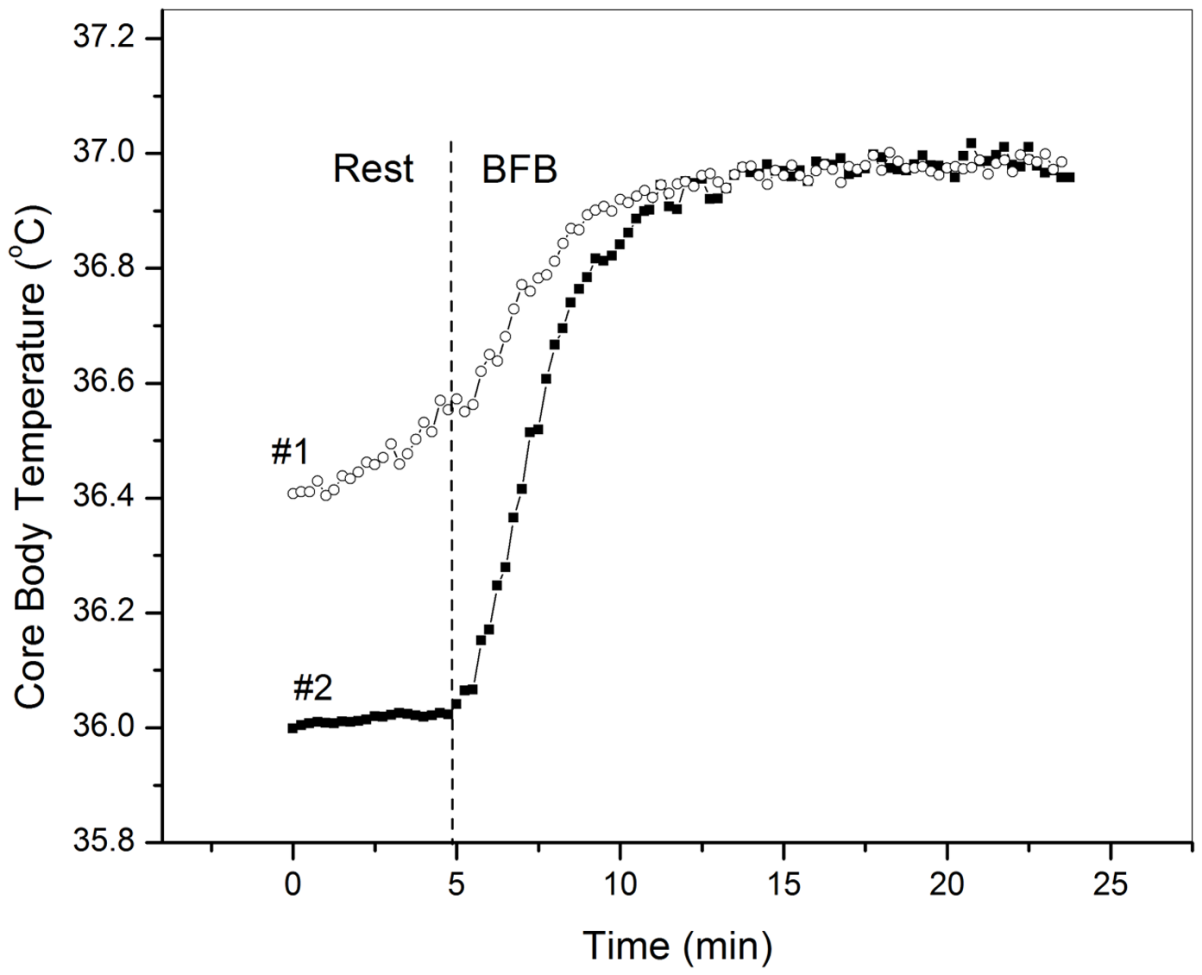
CBT increases during baseline and BFB in Study 2.

The CBT changes during baseline (rest) and BFB for two representative participants are shown in **Figure 6**. Similar to Study 1, in order to quantify the effective period of steady temperature increase during BFB before equilibrium was reached we used the *rise time* (ΔTr) as the time taken for the CBT to rise from 10% to 90% of its final value. To quantify the rate of CBT increase, we conducted linear regression analyses (CBT increase was regressed on the overall rest time for baseline period and on the rise time for the BFB practice) for each participant separately. All linear regressions for each participant for each of the above periods were significant (all R squares >0.92; *p*s <0.001). Furthermore, the linear mixed effect (MIXED) model was conducted to estimate the population regression slopes representing the rate of axillary temperature changes. The estimated regression slopes were δ = 0.006°C/min for the baseline period before BFB and δ = 0.091°C/min for the period of BFB practice. The regression slopes were significantly different from zero for the BFB time period only (*p*<0.05).

Although the estimated regression slope was not significantly different from zero for the baseline period (and in fact, 7 participants showed relatively constant CBT during the baseline, such as participant #2, Fig. 6), there were four participants who exhibited some noticeable gradual increase of their CBT during the rest (e.g., participant #1, Fig. 6). The average rate of the CBT increase for these four participants during this time period was δ = 0.009°C/min, which is comparable to the rate of CBT increase of up to 0.5°C/hour due to CBT circhoral rhythmic fluctuations [21], [26]. Thus, the observed increases during baseline rest seem to reflect daily CBT oscillations. The rate of these CBT increases, however, is 10 times less than the rate of CBT increases observed during BFB practice.

The increases in CBT during BFB were significant [paired-samples, two-tailed *t*(10) = 3.196, *p* = 0.01]. The average initial CBT of all the participants before BFB was 36.38°C (SD = 0.23), while the average temperature at the end of BFB reached 36.99°C (SD = 0.13), only marginally above the normal axillary temperature of 36.6°C in the healthy population [one sample two-tailed *t*(10) = 2.02, *p* = 0.07]. The maximum CBT reached during BFB was 37.02°C. The average apnea duration of the participants was 30.38 sec (SD = 6.35), ranging from 19 to 41 sec; the apnea duration correlated with the rate of CBT increase during BFB: *r* = 0.60, *p* = 0.050. Independent sample *t*-tests (two-tailed) conducted to compare the CBTs of the Western participants in Study 2 with those of 6 Tibetan meditators in Study 1 before and after BFB revealed no significant differences between the two groups’ initial CBTs, *t*(15) = 0.85, *p* = 0.40 or their CBT at the end of the BFB, *t*(15) = 0.39, *p* = 0.69. However, the final CBTs reached by the Tibetan practitioners at the end of *MFB* were significantly higher than the final CBT of the Western participants at the end of BFB, *t*(15) = 3.92, *p* = 0.001.

The cool-down phase started immediately after completion of BFB (average rate of cool-down is δ = 0.034°C/min); all the Western non-meditator participants returned to their baseline CBT during the next 20 minutes. This is in contrast to Tibetan practitioners performing GB (Study 1) who did not show any decreases in their CBTs during either BGB or MGB (δ = 0.011°C/min and δ = 0.002°C/min respectively), and were able to maintain an elevated body temperature throughout the whole duration of MGB (on average 21.50 min). This further confirms that the GB practice facilitates maintenance of body temperature.

**Figure 7:**
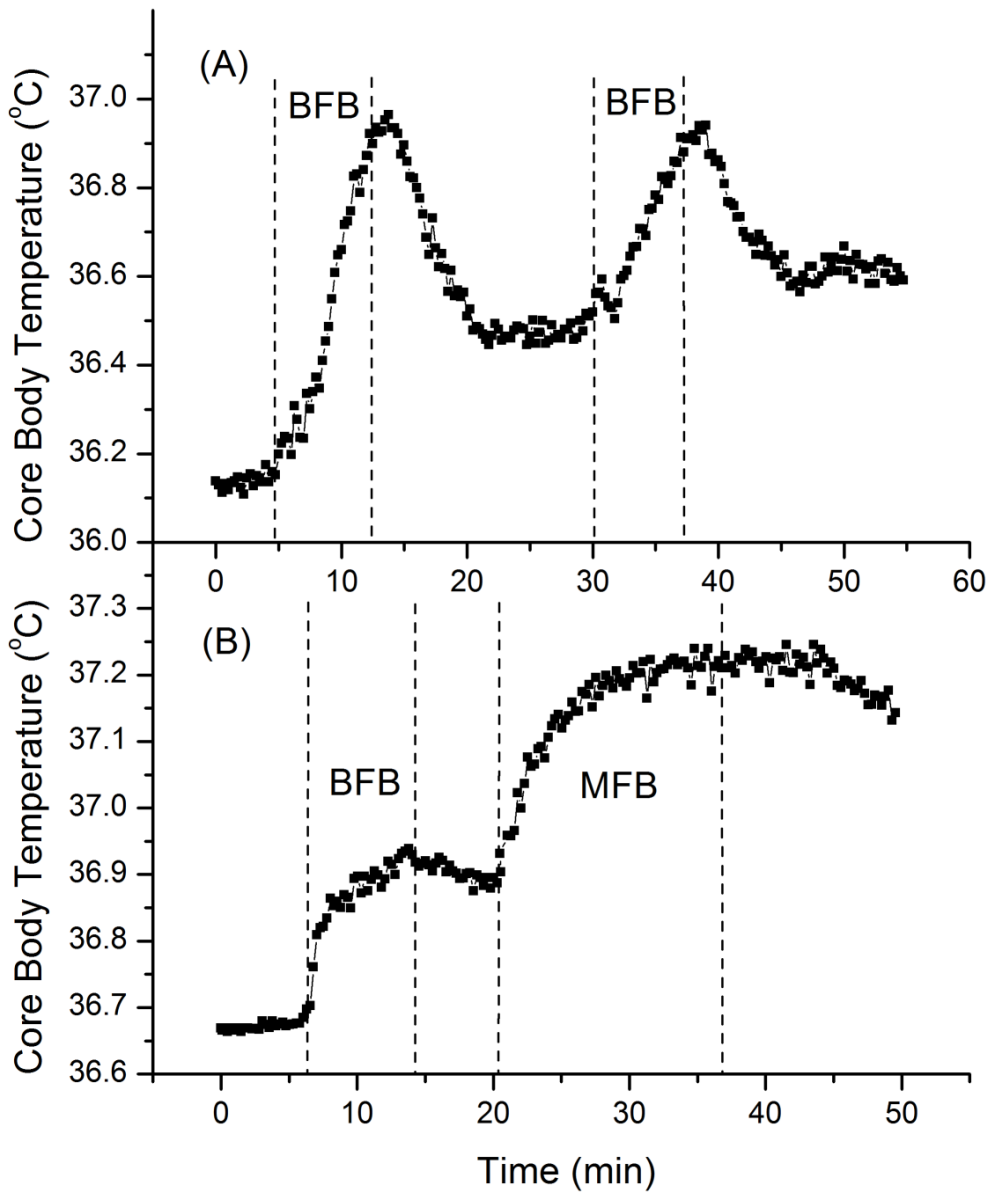
CBT increases during FB as performed by a Western non-meditator and a Western g-tummo practitioner. The vertical dashed lines indicate the beginning/end of different phases of the practice; (A, Western non-meditator): rest, BFB, rest, BFB, rest, and (B, Western g-tummo pract itioner): rest, BFB, rest, MFB, rest.

**Figure 7A** presents data from the Western participant who performed BFB twice. As can be seen in the figure, the participant raised her CBT to 36.96°C during the first BFB session, then stopped when she felt uncomfortable, and after about 10 min rest (accompanied by a gradual decrease in CBT), she started the second session of BFB. However, she was not able to raise her CBT any higher during the second BFB session, even though she started the session at a higher baseline temperature. This contrasts with the Western g-tummo practitioner who performed BFB followed by MFB (**Figure 7B**). This participant reached a similar CBT of 36.95°C by the end of the BFB session, but was able to raise her CBT further during MFB up to the zone of slight fever (37.03°C).

In summary, the results of Study 2 indicate that the BFB technique brings about significant increases in CBTs not only in meditators but also in those individuals who do not have any prior experience in meditation. The rate of these increases is much higher than the rate of the temperature increases due to CBT circhoral rhythmic fluctuations. However, the overall CBT increases due to the BFB technique are limited and in the range of normal body temperature. The data further suggest that the meditative visualization involved in MFB allows the practitioner to reach CBTs higher than can be reached using breathing and isometric techniques alone.

## Discussion

The findings of our research indicate that there are two distinct types of g-tummo practice, FB and GB, each characterized by different temperature patterns and neural (EEG) correlates. The temperature data bear out the practitioners’ claims that FB meditation is used to increase body heat, while GB meditation seems to facilitate the maintenance of an elevated body temperature. Indeed, reliable CBT increases were observed during the FB component of g-tummo practice, not only via FB meditation but also via FB vase breathing alone. However, the results of Studies 1 and 2 also suggest that that the neurocognitive component (“internalized attention” on visual images) of the MFB practice may facilitate elevation in CBT beyond the range of normal body temperature (into the fever zone), whereas the CBT increases during FB vase breathing alone were limited, and did not exceed the range of normal body temperature.

The results of the EEG analysis also indicate that FB and GB meditation were associated with different brain states in the practitioners. The EEG data showed significant increases in alpha and beta, and a marginally significant increase in gamma power during MFB, whereas during MGB only beta increased. Although the potential functional role of beta-band oscillations is not yet well understood, beta activity is associated with focused executive attention, and it seems to be related to the maintenance of one’s current sensorimotor or cognitive state [27]. Thus, significant increases in beta, as observed in the present research as well as in the study by Benson et al [7] might correspond to enhancement of attentional processes during both FB and GB meditation compared to their corresponding baselines. As for the increased gamma activity observed during FB meditation, studies on meditation consider it a signature of “samadhi” (deep meditative states of consciousness), but the regions of increase have varied, with recent studies reporting in some cases a frontally distributed increase in gamma [28], and in other cases an increase in gamma at posterior and occipital electrodes [29]. Our data showed that gamma had the highest power at frontal areas during the FB meditation (however, the frontal distribution of gamma effects should be considered with caution as these high frequency changes might be confounded with activity of supraficial muscles and eye movements [30].

Of particular interest is the increase in the power of the alpha frequency band which was observed during FB meditation and its significant relation to the CBT increases. Contrary to an early view that alpha represented an idling process in the visual system, evidence has begun to accumulate in support of a role for alpha oscillations as a general inhibitory mechanism in the brain [31], [32]. Specifically, increased alpha activity in occipito-parietal areas has been linked to the successful suppression of visual input, which could disturb the maintenance of visual working memory representations [33], [34]. This is consistent with a more general observation that an inward shift of attentional focus toward mental activity (as in mental rotation or other visual-spatial imagery tasks) is typically accompanied by increases in posterior alpha power [35], [36], suggesting that alpha might be working to decrease the distractibility of “external” sensory events to aid concentration on the mental activity. Similarly, meditation research has suggested that increases in alpha power correspond to “internalization of attention” (internally directed attention) during task performance as compared to baseline [37], which seems to be a plausible interpretation of our results. During FB meditation, practitioners are supposed to direct their attention to internally generated sensory information (visual imagery, heat perception). In contrast, there were no significant increases in alpha power during vase breathing alone, suggesting that these increases could be specifically attributed to the meditative component of the practice. We suggest that the increase in alpha power observed in our study reflects the meditators’ ability to focus attention on relevant internal states, specifically on visual images of flames and sensations of heat, while inhibiting external sensory information for extended periods.

The central finding of our study is that there are at least two separate factors that affect CBT increases during FB meditation. The first factor is related to the somatic component of the FB practice, specifically to the effectiveness of the vase breathing technique that affects the rate of CBT increase. Indeed, the results of studies 1 and 2 showed a significant correlation between the rate of CBT increases and apnea duration, during which the practitioners hold their breath while concurrently maintaining isometric muscle tension. The rate of these CBT increases was significantly higher than that due to circhoral rhythmic fluctuations in CBT, and it was similar to the rate of CBT increases of 0.02–0.17°C per min during the use of noninvasive systemic hyperthermia methodologies (e.g., immersing a patient’s body in hot water or wax, wrapping the body in a blanket or suit through which heated water is pumped, irradiating with IR energy) [22]. The second factor is related to the neurocognitive component of the FB practice, specifically to the amount of “internalization of attention” or quality of meditative visualization, as reflected by increased alpha power during FB meditation. This factor seems to determine the *rise time* of CBT, that is, how long the meditators are capable of continuing to raise their CBT beyond the range of normal body temperature without reaching an equilibrium phase. Indeed, the results of Study 1 showed that the greater the increase in alpha power achieved during FB meditation, the longer the CBT rise time, leading to higher CBTs.

Both factors work in conjunction to maximize the temperature increase. That is, the FB somatic component (vase breathing) causes thermogenic effects, while the neurocognitive component (meditative visualization) seems to be key for facilitating a sustained increase in body temperature for longer periods, possibly due to mitigating physiological mechanisms leading to heat loss. In systemic hyperthermia treatments, to prevent heat loss, and thus sustain CBT increases for longer periods, different insulation techniques are used (e.g., wrapping a patient body in reflective blankets, foil, or plastic films [22]). In the case of FB meditation, one of the possible mechanisms preventing heat loss could be the mental imagery of flames and heat. Indeed, previous research has regarded mental imagery as a potentially effective technique in influencing peripheral body temperature, blood flow, and local vasodilation [38]–[41]. Thus, it is possible that the mental imagery component of FB meditation minimizes heat loss, and thus prolongs the CBT rise time by similar mechanisms (changes in blood flow, reduced vasodilation). Without the accompanying meditative visualization, vase breathing might not be very effective and result in only limited CBT increases. At the same time, without an effective FB breathing technique, even small CBT increases, if possible at all, might require significantly longer meditation periods.

One of the questions arising from this study is why breathing and isometric exercises have been chosen by Tibetan meditators as a mean of thermogenesis, instead of dynamic movements (e.g., physical exercising, running). Different types of breathing and isometric techniques have been used for thousand years not only in Tibetan traditions but also in static holds in certain branches of yoga or oriental martial arts. What is common to all these practices is that they require focused attention on the internal mental states, which is difficult to do during rigorous dynamic exercise. Indeed, according to the g-tummo practitioners’ reports, FB meditation requires significant concentration, and cannot be performed in conjunction with running or even walking. For these reasons, during the ceremony of drying wet sheets, while walking outside, g-tummo meditators do not perform FB, but GB meditative visualization, which is much less effortful.

A limitation affecting the generalizability our findings is the small sample size due to the sacredness of the practice and difficulties in accessing g-tummo practitioners. Despite this limitation, we were able, for the first time, to document reliable CBT increases during the FB type of g-tummo practice, all within the slight to moderate fever zone, validating the legends of the extraordinary capacity of g-tummo meditators to elevate their body temperature beyond normal. However, the results also suggest that temperature increases during g-tummo meditation are neither solely a by-product of meditation nor its goal, but instead may be a *means* to facilitate the achievement of “deep meditative states”. The g-tummo meditators may use the CBT increases as a vehicle to enhance their attention and focus their meditative performance (which may in turn facilitate a further increase in their temperature through meditative visualization). Future studies of experts in g-tummo meditation who are capable of elevating and maintaining elevated CBT may offer promising research insights and approaches to investigating mechanisms of CBT regulation. Because many variables underlying neuronal functioning (e.g., transport via ion-selective channels, amplitude and duration of single-unit spikes) are temperature-dependent [42], [43], possibility of self-regulation of CBT may have a direct effect on self-regulating and maximizing neurocognitive activity. If future studies show that it is possible to self regulate CBT, by mastering vase breathing in conjunction with guided mental imagery without extensive meditation experience, it will open a wide range of possible medical and behavior interventions, such as adapting to and functioning in hostile (cold) environments, improving resistance to infections, boosting cognitive performance by speeding response time, and reducing performance problems associated with decreased body temperature as reported in human factor studies of shift work and continuous night operations [44], [45].

## References

[1] Evans-Wentz WY (2002) Tibetan Yoga and Secret Doctrines (Pilgrims Publishing, Varanisa, India).

[2] Mullin GH (1997) Readings on Six Yogas of Naropa (Snow Lion Publication, Ithaca, NY).

[3] Mullin GH (1996) Tsongkhapa’s Six Yogas of Naropa (Snow Lion Publication, Ithaca, NY).

[4] David-Neel A (1971) Magic and Mystery in Tibet (Dover Publications, New York).

[5] Govinda Lama Anagarika (1988) Way of White Clouds (Shambhala Publications,).

[6] Benson H, Lehmann JW, Malhotra MS, Goodman RF, Hopkins J, et al (1982) Body temperature changes during the practice of g-tummo yoga. Nature 295: 234–236. 7035966 10.1038/295234a0

[7] Benson H, Malhotra MS, Goldman RF, Jacobs GD, Hopkins PJ (1990) Three case reports of the metabolic and electroencephalographic changes during advanced Buddhist meditation techniques. Behav. Med 16: 90–95. 10.1080/08964289.1990.9934596 2194593

[8] Cromie WJ (2002) Meditation changes temperatures: Mind controls body in extreme experiments. Harvard University Gazette.

[9] Bird EI, Colborne GR (1980) Rehabilitation of an electrical burn patient through thermal biofeedback. Biofeedback Self. Regul 5: 283–288. 10.1007/BF00998604 6156713

[10] Willerman L, Skeen JT, Simpson JS (1976) Retention of learned temperature changes during problem solving. Percept. Mot. Skills 43: 995–1002. 10.2466/pms.1976.43.3.995 1012880

[11] Taub E, Emurian CS (1976) Feedback-aided self-regulation of skin temperature with a single feedback locus: I. Acquisition and reversal training. Biofeedback Self. Regul I: 147–168. 10.1007/BF00998583 990346

[12] King NJ, Montgomery RM (1981) The self-control of human peripheral (finger) temperature: An exploration of somatic manoeuvres as aids to biofeedback training. Behav. Ther 12: 263–27.

[13] Singh V, Aballay A (2006) Heat-shock transcription factor (HSF)-1 pathway required for Caenorhabditis elegans immunity. Proc Natl Acad Sci USA 103: 13092–13097. 16916933 10.1073/pnas.0604050103 PMC1559758

[14] Kluger MJ, Kozak W, Conn CA, Leon LR, Soszynski D (1996) The adaptive value of fever. Infect. Dis. Clin. North. Am 10: 1–20. 10.1016/s0891-5520(05)70282-8 8698984

[15] Mackowiak PA (1994) Fever: Blessing or Curse? A Unifying Hypothesis Ann. Intern. Med June 15: 10371040. 10.7326/0003-4819-120-12-199406150-00010 7832829

[16] Masino SA, Dunwiddie TV (2000) A transient increase in temperature induces persistent potentiation of synaptic transmission in rat hippocampal slices. Neuroscience 101: 907–912. 11113339 10.1016/s0306-4522(00)00431-0

[17] Kleitman N, Jackson DP (1950) Body temperature and performance under different routines. J. Appl. Physiol 3: 309–328. 10.1152/jappl.1950.3.6.309 14794593

[18] Holland RL, Sayer JA, Keatinge WR, Davis HM, Peswani R (1985) Effects of raised body temperature on reasoning, memory, and mood. J. Appl. Physiol 59: 1823–1827. 10.1152/jappl.1985.59.6.1823 4077790

[19] Wright KP Jr, Hull JT, Czeisler CA (2002) Relationship between alertness, performance and body temperature in humans. Am. J. Physiol. Regul. Integr. Comp. Physiol 283: R1370–R1377. 10.1152/ajpregu.00205.2002 12388468

[20] Kelly G (2006) Body temperature variability (Part 1): a review of the history of body temperature and its variability due to site selection, biological rhythms, fitness, and aging. Altern. Med. Rev 11: 278–93. 17176167

[21] Mackowiak PA, Wasserman SS, Levine MM (1992) A critical appraisal of 98.6 degrees F, the upper limit of the normal body temperature, and other legacies of Carl Reinhold August Wunderlick. J. Am. Med. Assoc 268: 1578–1580. 1302471

[22] Rowe-Horwege RW (2006) Hyperthermia, Systemic. Encyclopedia of Medical Devices and Instrumentation.

[23] Levine WS (1996) The control handbook. Boca Raton, FL: CRC Press.

[24] Nise NS (2008) Control Systems Engineering (Fifth ed). John Wiley & Sons.

[25] Kiebel SJ, Tallon-Baudry C, Friston KJ (2005) Parametric analysis of oscillatory activity as measured with EEG/MEG. Hum. Brain Mapp 26: 170–177. 10.1002/hbm.20153 PMC6871741 15929085

[26] Lindalwy G, Dowse HB, Burgoon PW, Kolka MA, Stephenson LA (1999) Persistent circhoral ultradian rhythm is identified in human core temperature. Chronobio. Internat (16) 69–78. 10.3109/07420529908998713 10023577

[27] Engel AK, Fries P (2010) Beta-band oscillations – signalling the status quo. Curr. Opin. Neurobiol 20: 156–165. 10.1016/j.conb.2010.02.015 20359884

[28] Lutz A, Greischar LL, Rawlings NB, Ricard M, Davidson RJ (2004) Long-term meditators self-induce high-amplitude gamma synchrony during mental practice. Proc. Natl. Acad. Sci USA 101 16369–16373. 10.1073/pnas.0407401101 PMC526201 15534199

[29] Cahn BR, Delorme A, Polich J (2010) Occipital gamma activation during Vipassana meditation. Cogn. Process 11: 39–56. 10.1007/s10339-009-0352-1 PMC2812711 20013298

[30] Whitham EM, Pope KJ, Fitzgibbon SP, Lewis T, Clark CR, et al (2007) Scalp-electrical recording during paralysis: quantitative evidence that EEG frequencies above 2-Hz are contaminated by EMG. Clin. Neurophysiol 118: 1877–1888. 10.1016/j.clinph.2007.04.027 17574912

[31] Klimesch W, Sauseng P, Hanslmayr S (2007) EEG alpha oscillations: the inhibition-timing hypothesis. Brain Res. Rev 53: 63–88. 10.1016/j.brainresrev.2006.06.003 16887192

[32] Jensen O, Mazaheri A (2010) Shaping functional architecture by oscillatory alpha activity: gating by inhibition. Front. Hum. Neurosci 4: 186. 10.3389/fnhum.2010.00186 PMC2990626 21119777

[33] Worden MS, Foxe JJ, Wang N, Simpson GV (2000) Anticipatory biasing of visuospatial attention indexed by retinotopically specific alpha-band electroencephalography increases over occipital cortex. J Neurosci 20: RC63. 10704517 10.1523/JNEUROSCI.20-06-j0002.2000 PMC6772495

[34] Sauseng P, Klimesch W, Gerloff C, Hummel FC (2009) Spontaneous locally restricted EEG alpha activity determines cortical excitability in the motor cortex. Neuropsychologia 47: 284–288. 18722393 10.1016/j.neuropsychologia.2008.07.021

[35] Jensen O, Gelfand J, Kounios J, Lisman JE (2002) Oscillations in the alpha band (9–12 Hz) increase with memory load during retention in a short-term memory task. Cereb. Cortex 12: 877–882. 10.1093/cercor/12.8.877 12122036

[36] Cooper NR, Croft RJ, Dominey SJJ, Burgess AP, Gruzelier JH (2003) Paradox lost? Exploring the role of alpha oscillations during externally vs. internally directed attention and the implications for idling and inhibition hypotheses. Int. J. Psychophysiol 47: 65–74. 10.1016/s0167-8760(02)00107-1 12543447

[37] Aftanas LI, Golocheikine SA (2001) Human anterior and frontal midline theta and lower alpha reflect emotionally positive state and internalized attention: High resolution EEG investigation of meditation. Neurosci. Lett 310: 57–60. 10.1016/s0304-3940(01)02094-8 11524157

[38] McGuik J, Fitzgerald D, Firedman PS, Oakley D, Salmon P (1998) The effect of guided imagery in a hypnotic context on forearm blood flow. Contemp Hypn 15: 101–108.

[39] Lee LH, Olness K (1996) Effect of self-induced mental imagery on autonomic reactivity in children. J Dev Behav Pediatr 17: 323–327. 8897220 10.1097/00004703-199610000-00006

[40] Serra D, Parris CR, Carper E, Homel P, Fleishman SB, et al (2012) Outcomes of guided imagery in patients receiving radiation therapy for breast cancer. Clin J Oncol Nurs 16: 617–23. 23178354 10.1188/12.CJON.617-623

[41] Moore LE, Wiesner SL (1996) Hypnotically induced vasodilation in the treatment of repetitive strain injuries. Am J Clin Hypn 39: 97–104. 8936710 10.1080/00029157.1996.10403372

[42] Thompson SM, Musakawa LM, Rince DA (1985) Temperature dependence of intrinsic membranes properties and synaptic potentials in hippocampal CA1 neurons in vitro. J. Neurosc 5: 817–824. 10.1523/JNEUROSCI.05-03-00817.1985 PMC6565032 3973697

[43] Erickson CA, Jung MW, McNaughton BL, Barnes CA (1996) Contribution of single-unit spike waveform changes to temperature induced alterations in hippocampal population spikes. Exp. Brain. Res 107: 348–360. 10.1007/BF00230417 8821377

[44] Colquhoun WP, Folkard S (1985) in Hours of Work: Temporal Factors in Work-Scheduling, eds Folkard S, Monk TH (Wiley, New York), 253–261.

[45] Campbell SS (1995) Effects of timed bright-light exposure on shift-work adaptation in middle-aged subjects. Sleep 18: 408416. 10.1093/sleep/18.6.408 7481411

